# Comparison of the *in Vitro* Uptake and Toxicity of Collagen- and Synthetic Polymer-Coated Gold Nanoparticles

**DOI:** 10.3390/nano5031418

**Published:** 2015-08-27

**Authors:** Oana T. Marisca, Karsten Kantner, Christian Pfeiffer, Qian Zhang, Beatriz Pelaz, Nicolae Leopold, Wolfgang J. Parak, Joanna Rejman

**Affiliations:** 1 Fachbereich Physik, Philipps Universität Marburg, Marburg 35037, Germany; E-Mails: oanamarisca@gmail.com (O.T.M.); kantnerk@staff.uni-marburg.de (K.K.); pfeiffer-christian@gmx.net (C.P.); qian.zhang@physik.uni-marburg.de (Q.Z.); beatriz.pelazgarcia@physik.uni-marburg.de (B.P.); wolfgang.parak@physik.uni-marburg.de (W.J.P.); 2 Faculty of Physics, Babeş-Bolyai University, Cluj-Napoca 400084, Romania; E-Mail: nicolae.leopold@phys.ubbcluj.ro; 3 CIC Biomagune, San Sebastian 20009, Spain; 4 Dr. von Hauner Children’s Hospital, Ludwig Maximilians University, Munich 80337, Germany

**Keywords:** gold nanoparticles, cellular uptake, toxicity, biocompatibility

## Abstract

We studied the physico-chemical properties (size, shape, zeta-potential), cellular internalization and toxicity of gold nanoparticles (NPs) stabilized with the most abundant mammalian protein, collagen. The properties of these gold NPs were compared to the same sized gold NPs coated with synthetic poly(isobutylene-*alt*-maleic anhydride) (PMA). Intracellular uptake and cytotoxicity were assessed in two cell lines (cervical carcinoma and lung adenocarcinoma cells) by employing inductively-coupled plasma-mass spectrometry (ICP-MS) analysis and a cell viability assay based on 3-(4,5-dimethylthiazol-2-yl)-2,5-diphenyltetrazolium bromide (MTT), respectively. We found that the collagen-coated gold NPs exhibit lower cytotoxicity, but higher uptake levels than PMA-coated gold NPs. These results demonstrate that the surface coating of Au NPs plays a decisive role in their biocompatibility.

## 1. Introduction

The development of new biocompatible nano-materials is an exponentially growing field of research. For example, the number of potential biomedical applications for gold nanoparticles (Au NPs) has expanded impressively in recent years. A vast part of this research is dedicated to the synthesis of Au NPs that could be employed in various medical fields [1], such as theranostics. They have been proposed as delivery systems to target drugs to diseased cells, tissues and organs and as contrast agents to enhance imaging in time-resolved optical tomography. Moreover, they can be combined with Raman reporters for detection purposes using surface-enhanced Raman scattering (SERS) [2,3]. For many of these applications, it is essential that the carrier system is biocompatible. The biocompatibility of NPs not only depends on their physicochemical properties associated with the bulk of the NPs, such as size or shape, but also on the properties associated with the type of material used for their surface coating, such as the zeta-potential [4]. Although the inertness of gold as such is in general not questioned, the stabilizing coating required to prevent aggregation of Au NPs might induce significant toxicity. As has been demonstrated extensively for other particulate drug systems, such as liposomes, a very important phenomenon governing the fate of Au NPs in living cells, as well as in cell culture systems is the adsorption of a protein layer (“corona”) on the NP surface. Depending on the NP size, shape and charge, a variety of different proteins might adsorb and, thus, determine not only the degree and mode of uptake and toxicity, but also the types of cells involved in the uptake and/or elimination process [5,6,7].

In this study, we compared two Au NP systems differing in surface coating in terms of their interaction with cells and culture media. One system was coated with a synthetic polymer, dodecylamine-modified poly(isobutylene-*alt*-maleic anhydride) (PMA) [8,9], and the other with a protein, collagen. The coated Au NPs were characterized with spectroscopic and microscopic techniques. To assess how the surface coating affects cell-NP interactions, internalization and cytotoxicity tests were performed in two cancer cell lines: cervical carcinoma (HeLa) and lung adenocarcinoma (A549) cells. The uptake of Au NPs was quantitatively evaluated by inductively-coupled plasma-mass spectrometry (ICP-MS), and intracellular NP localization was visualized with fluorescence microscopy. Cell viability was probed with the 3-(4,5-dimethylthiazol-2-yl)-2,5-diphenyltetrazolium bromide (MTT) assay [10,11].

## 2. Materials and Methods

### 2.1. Synthesis of Collagen-Coated Au NPs

Collagen has both a reducing and a stabilizing role in Au NP formation. A stock solution of gold salt was prepared by dissolving 1 g hydrogen tetrachloroaurate (III) hydrate (99.9% metal basis, Alfa Aesar, Karlsruhe, Germany) in 50 mL ultrapure water. A solution of collagen was prepared by mixing 10 mL ultrapure water with 0.02 g of collagen from bovine Achilles tendon (Sigma-Aldrich, Darmstadt, Germany) in the presence of 500 µL hydrochloric acid 37% (Sigma-Aldrich). One-point-five milliliters of collagen solution (0.02 g collagen in 10 mL water) was mixed with 0.5 mL ethanol. Subsequently, 90 mL of ultrapure water were added to the collagen-ethanol solution, which was then mixed with 1 mL of gold salt solution. The mixture was heated and stirred until boiling. When it started to boil, the solution was neutralized by quickly adding 2 mL of 1% sodium hydroxide, and the heating was turned off. Instantly, the solution turned into a wine-red color, and its pH was 7. Sodium hydroxide (Sigma-Aldrich), ethanol and sodium chloride (Merck, Darmstadt, Germany) were of analytical grade. All solutions were prepared in ultrapure water with a resistance higher than 18 MΩ (Direct-Q 3 UV, Merck Millipore, Darmstadt, Germany). The protocol is given in full detail in the Supporting Information. The Raman spectrum of collagen-coated Au NPs is presented in Figure S1.

### 2.2. Fluorescence-Labelling of Collagen-Coated Au NPs

Collagen-coated Au NPs were filtered three times and then suspended in bicarbonate buffer pH = 8.6. Their concentration was calculated from the UV-VIS absorption spectrum (molar extinction coefficient ε = 2.03 × 10^7^ M^−1^·cm^−1^) [12]. One milligram of Dy™647 (Dyomics, Jena, Germany, absorption/emission max 653/672 nm) was dissolved in 1 mL of bicarbonate buffer pH = 8.6. Then, 200 µL collagen Au NPs (2.8 µM) were added to 750 µL dye solution in an Eppendorf tube and allowed to mix for 3 h. The mixture of the dye and the collagen Au NPs was then filtered in 5 steps, and the NPs were suspended in water. After sterile filtration, again, the UV-VIS absorption spectrum of labelled collagen Au NPs was recorded. The uptake of collagen Au NPs by HeLa cells was visualized by fluorescence microscopy.

### 2.3. Synthesis of PMA-Coated Au NPs

Hydrogen tetrachloroaurate (III) hydrate (Alfa Aesar), sodium borohydride (Sigma-Aldrich) and tetraoctylammonium bromide (TOAB; Sigma-Aldrich) were used to synthesize Au NPs (4–5 nm in diameter) according to previously published protocols [9,13]. Briefly, an aqueous solution of HAuCl_4_ was transferred to toluene to form an ionic pair with TOAB (4.5 eq.), which also acts as a stabilizing agent. In the organic phase, sodium borohydride (NaBH_4_, 10 eq.) was added to reduce Au^3+^ to Au^0^, leading to the formation of colloidal Au NPs. The NPs were then washed to remove the excess of ions with HCl, NaOH and, finally, Milli-Q water. Each time, the added aqueous phase was discarded. Ostwald ripening, facilitating the formation of a monodisperse suspension, occurred during an overnight incubation. 1-dodecanethiol was added to replace the surfactant ligands on the particle, rendering the NPs more stable. NPs were further purified with methanol and finally re-dispersed in chloroform. It must be noted that upon ligand exchange of TOAB to 1-dodecanethiol, part of the original TOAB molecules may be left on the Au surface. Ligand exchange processes are known to be not always 100% complete [14]. A detailed analysis of the molecules present on the surface, e.g., by mass spectroscopy, is complicated, due to the presence of several molecular species. A detailed examination is currently under way. After ligand exchange, the NPs were washed several times. The hydrophobic Au NPs were then transferred into an aqueous solution by coating them with 1-dodecylamine-modified poly(isobutylene-alt-maleic anhydride (PMA, Sigma, Darmstadt, Germany; *M*_w_ = 6000 g mol^−1^), as described previously [8,9,15,16]. For the coating procedure, the following parameters referring to previous publications were used: molar extinction coefficient of the Au NPs ε = 1.14 × 10^7^ M^−1^·cm^−1^, core diameter *d*_c_ = 4.4 nm, thickness of surfactant layer *l* = 1.2 nm, leading to the effective diameter *d*_eff_ = 6.8 nm, number of added polymer monomer units per effective NP surface area *R*_P/area_=200 nm^−2^. Seventy five percent of the anhydride rings of the polymer were linked with dodecylamine. Optionally, the fluorophore Dy647 was incorporated into the polymer, as described in previous reports [17]. Hereby, 1% of the anhydride rings were linked with fluorophore.

### 2.4. Nanoparticle Purification and Characterization

Both types of NPs were filtered and then purified by gel electrophoresis (*cf.* Supporting Information Figures S2 and S3). Two percent gels were prepared by adding 7 g of agarose in 350 mL of 0.5 × TBE buffer (Tris/borate/EDTA, pH 8.3) [18]. This mixture was brought to boiling, and one-well gels were prepared. The Au NPs were run in the gels for 60 min, with an applied voltage of 10 V/cm. Gel parts containing the NPs were cut out and placed in a dialysis membrane (50-kDa molecular weight cut-off (MWCO); Spectrum Labs Europe, Breda, The Netherlands) filled with 0.5 × TBE buffer, which was then exposed for 15 min to the same voltage in order to elute the NPs, which were then collected inside the dialysis membrane tubes. The extracted NPs were filtered and concentrated in water via ultrafiltration (100-kDa MWCO membranes). Prior to the experiments, the NPs were filter-sterilized (0.02 μm), and their concentration was evaluated by UV-VIS spectroscopy (molar extinction coefficient ε as given above). UV-VIS absorption spectra of the prepared gold colloids were recorded on a Jasco V-630 UV-VIS spectrophotometer, by using quartz cuvettes with an optical path length of 1 cm (*cf.* Supporting Information Figure S4). The size and morphology of the Au NPs were determined using transmission electron microscopy (TEM) imaging performed with a JEOL Model JEM 1010 microscope, JEOL Germany GmbH, Freising, Germany). High resolution TEM micrographs of the Au NPs were recorded with a PHILIPS CM 20 microscope (Eindhoven, The Netherlands) operated at 200 kV. The diameters of the inorganic cores *d*_c_ of the NPs were calculated from the TEM micrographs using the ImageJ 1.45s software from Wayne Rasband, NIH, USA. The hydrodynamic diameter, *d*_h_, and the zeta potential, ζ, of Au NPs were assessed with a Malvern Zeta-sizer (Malvern Instruments Ltd, Worcestershire, UK), based on dynamic light scattering (DLS) and laser Doppler anemometry (LDA), respectively.

### 2.5. Cell Culture

Cervical carcinoma (HeLa) and adenocarcinoma alveolar basal epithelial (A549) cells were cultured in Dulbecco’s Modified Eagle’s culture medium (DMEM, Sigma-Aldrich) supplemented with 2 mM glutamine (Sigma-Aldrich), 10% FBS (fetal bovine serum) and 100 U/mL penicillin/streptomycin (Sigma-Aldrich). The cells were grown at 37 °C in a humidified atmosphere containing 5% CO_2_. Cells were seeded on 96-well plates at a density 5000 cells/well 24 h before experiments.

### 2.6. Particle Internalization and Sample Preparation for ICP-MS Measurements

A549 and HeLa cells were plated in 96-well plates at a density of 5000 cells/well 24 h prior to the experiments. The cells were incubated with different concentrations of Au NPs for 4 h. Au NPs were incubated with the cells in media with or without serum. The supernatants were collected and stored for further analysis at −20 °C. The cells were cultured for an additional 20 h. Then, the medium was removed, and the cells were lysed with a lysis buffer (Cell Lysis Buffer, Promega, Mannheim, Germany). After 30 min, the samples were collected and stored at −20 °C. Prior to ICP-MS measurements, 150 µL of 37% HCl were mixed with 50 µL of 65% HNO_3_ and after 5 min, added to each sample. This was followed by the addition of 2.7 mL of 2% HCl. This treatment ensured sample decomposition to near atom size and allowed for more accurate measurements [17].

### 2.7. Cell Viability

Toxicity induced by gold NPs was evaluated with the MTT assay [11]. The assay is based on the reduction of the tetrazolium salt MTT to formazan by metabolically-active cells. The produced formazan can be quantitatively evaluated by UV-VIS absorption spectroscopy due to its absorption maximum at 570 nm. Cells were plated in 96-well plates at a density of 5000 cells/well 24 h before experiments. Different concentrations of both types of Au NPs were incubated for 4 h with HeLa or A549 cells in the presence of serum. After removing the medium with the excess NPs, the cells were cultured for an additional 20 h. An MTT assay was then performed according to the manufacturer’s instructions (Roche, Basel, Switzerland; currently Sigma-Aldrich).

## 3. Results and Discussion

### 3.1. Synthesis and Characterization of Au NPs

**Figure 1:**
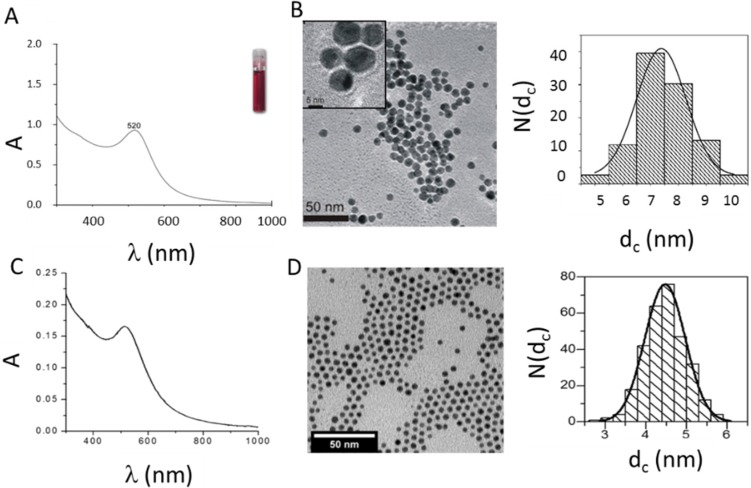
(**A**) UV-VIS absorption spectrum *A*(λ) of collagen-coated gold nanoparticles (Au NPs). The inset shows a vial with a solution of the Au NPs in water. (**B**) Transmission electron microscopy (TEM) images and size distribution of collagen-coated Au NPs. The scale bar in the inset corresponds to 5 nm and the scale bar in the main image to 50 nm. From the size distribution *N*(*d*_c_) of the inorganic cores, as seen in the TEM image, the mean value ± the standard deviation was determined to be *d*_c_ = 7.4 ± 2.4 nm. N corresponds to the number of counted NPs. (**C**) UV-VIS absorption spectrum *A*(λ) of PMA-coated Au NPs in water. (**D**) TEM images and size distribution *N*(*d*_c_) of PMA-coated Au NPs. The scale bar corresponds to 50 nm. As a result, *d*_c_ = 4.4 ± 1.1 nm was obtained.

We developed a synthesis procedure for Au NPs in which collagen acts both as a reducing and as a stabilizing agent. The one-step synthesis of protein-coated Au NPs prevents the use of reducing agents and stabilizers of a chemical nature. Thus, no surface modification step is required, and the synthesized Au NPs are exclusively covered by a protein layer, which in principle could be an advantage in terms of biocompatibility. In contrast to other techniques, in which Au NPs are generated free of ligands by laser ablation and then covered by adsorbed proteins [19], in the protocol introduced here, protein coating was directly achieved upon wet-chemistry synthesis routes. Most importantly, there is no other ligand than collagen present on the NP surface. As shown in Figure 1A, the collagen-coated Au NPs synthesized in this work are characterized by the presence of the typical plasmon resonance at 520 nm, which is commonly observed for Au colloids [20,21]. As demonstrated in Figure 1C, the same plasmon band position at 520 nm was observed for PMA-coated Au NPs. As no surface-enhanced Raman signal for collagen-coated Au NPs was observed (*cf.* Supporting Information, Figure S1), we assume an adsorption of collagen to Au NPs due to an electrostatic mechanism, similar to that proposed for albumin [22]. The TEM micrographs, shown in Figure 1B, demonstrate that the collagen Au NPs display a relatively uniform morphology of an approximately spherical shape with a mean diameter of the inorganic Au core of *d*_c_ = 7.4 nm, *i.e.*, excluding the contribution of the collagen coating to the size, which does not provide contrast in TEM [4].

The hydrodynamic diameter obtained by DLS from the number distribution in water and the zeta-potential were *d*_h_ ≈ 13.5 ± 0.5 nm and ζ = −58.4 ± 2.5 mV, respectively (the raw data are presented in Supporting Information Figure S5; the data for dye-modified NPs are in Figure S6). As a comparison, PMA-coated Au NPs, synthesized according to standard procedures [8,9], were used. These had plasmon resonance at 517 nm (Figure 1C), an inorganic core diameter of *d*_c_ = 4.4 nm (Figure 1D), a hydrodynamic diameter *d*_h_ ≈ 11.7 nm and a zeta potential ζ = −38 mV in water (Figure S5), respectively. The overall size in water (*d*_h_), as well as the zeta potentials (ζ) are similar for both types of NPs, which suggest similar physico-chemical properties of collagen and PMA Au NPs in water. The high negative zeta potential values indicate a good colloidal stability in water. However, the surface coating of both types of NPs is quite different, one being a protein and the other a synthetic polymer.

The colloidal properties of NPs, however, are strongly dependent on the medium in which they are dispersed. The presence of salt may screen the charge of the NPs, and proteins may be adsorbed to the NPs surface [4]. The colloidal properties of the NPs thus should be probed in the medium in which they are administered to cells. Different interactions with cells can be related to the changes of the colloidal properties in different media [23]. A convenient test involves the detection of the changes of the hydrodynamic diameter over time in the respective media. In the case that the hydrodynamic diameter significantly increases, agglomeration of the NPs can be assumed [24]. In Table 1, the time dependence of the hydrodynamic diameter of collagen- and PMA-coated Au NPs upon dispersion in cell medium without and with serum is shown (for the raw data, *cf.* Supporting Information Figure S7). The hydrodynamic diameter of PMA-coated Au NPs was found to be very stable in both types of culture media, and it did not change significantly over a period of 4 h. Thus, we may assume that the PMA-coated Au NPs are still well dispersed in cell media (*i.e.*, supplemented DMEM). Quite differently, collagen-coated Au NPs were stable in medium with serum, but were prone to form aggregates in medium without serum. Similar results were found for bovine serum albumin-coated Au NPs when exposed to comparable media [25,26]. Our data suggest that the collagen is only loosely bound to the surface of the Au NPs. As demonstrated in Table 1, the hydrodynamic diameter of collagen-coated Au NPs slightly increased in the presence of serum. We hypothesize that this could be caused by the adsorption of other proteins, which may provide further stability to the particles.

**Table 1:** Hydrodynamic diameter *d*_h_ of Au NPs dispersed in cell culture medium with or without serum, *t* = 0 h (*i.e.*, before), 2 h and 4 h after exposure to the medium. The concentration of Au NPs was *c*(NP) = 100 nM.

Type of Nanoparticle		*d*_h_ (nm)		
		*t*_0_	*t*_2h_	*t*_4h_
Collagen-Coated Au NPs	+ serum	18.1	18.1	18.1
Collagen-Coated Au NPs	− serum	13.5	141.8	220
PMA-Coated Au NPs	+ serum	8.7	8.7	8.7
PMA-Coated Au NPs	− serum	15.6	15.6	15.6

### 3.2. Cellular Internalization of NPs

**Figure 2:**
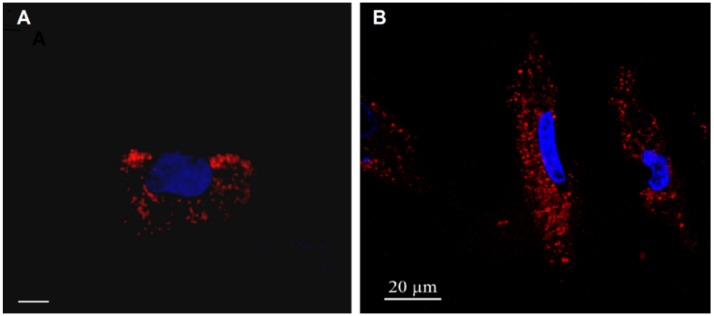
Internalization of Au NPs by HeLa cells. Images of HeLa cells incubated with fluorescence-labelled Au NPs. (**A**) Collagen-coated Au NPs; the scale bar corresponds to 10 µm. (**B**) PMA-coated Au NPs; the scale bar corresponds to 20 µm. The NPs were labelled with Dy^TM^647 and, thus, appear in read. The nuclei were labelled with DAPI and, thus, appear in blue.

We compared the uptake of both types of Au NPs by HeLa and A549 cells. First, the uptake of NPs was visualized with fluorescence microscopy. For this purpose, cells were incubated with Au NPs, which were labelled in their collagen or PMA shell with Dy647. In Figure 2, fluorescence microscopy images of cells after 4 h of incubation with the NPs are shown. In these qualitative images, it can be seen that the NPs (red fluorescence due to the Dy647) are located in grainy structures around the nucleus (blue fluorescence due to staining with 4',6-diamidino-2-phenylindole (DAPI)), which indicates for both types of NPs uptake via endocytosis and final localization in the endolysosomal compartment. As this is the expected classical pathway for NP internalization, no quantitative evaluation and localization studies were performed. Instead, NP uptake was quantified via ICP-MS.

**Figure 3:**
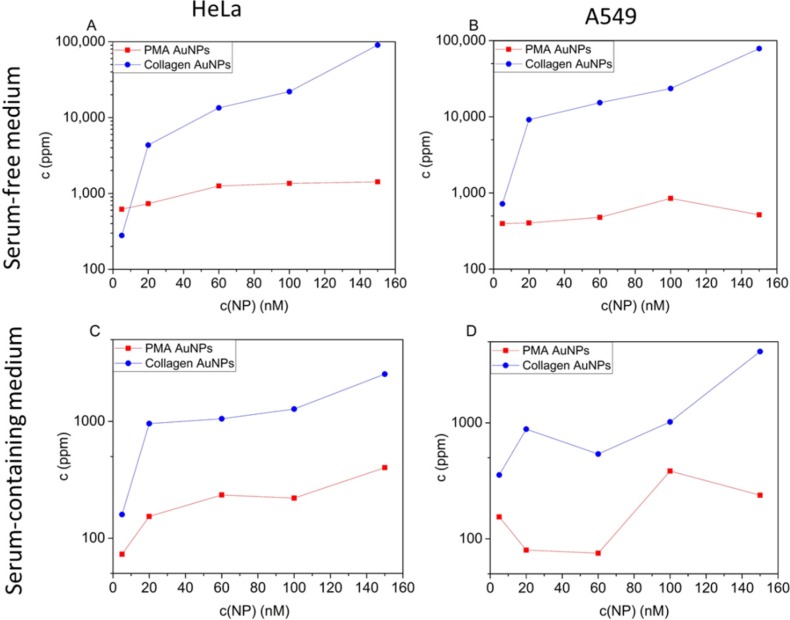
Internalization of Au NPs by cells. Collagen- and PMA-coated Au NPs were added to HeLa (**A**,**C**) or A549 (**B**,**D**) cells in medium without (**A**,**B**) and supplemented (**C**,**D**) with serum at different NP concentrations *c*(NP). After 4 h of incubation, the media with the excess of non-internalized NPs were removed, and the cells were thoroughly washed. The cells were cultured for an additional 20 h. The medium was then removed, and the cells were lysed. The samples were analyzed with ICP-MS, and the amount of internalized elemental gold *c*(Au) was determined. The lines between the individual points are merely intended as a guide to the reader.

For quantitative uptake studies, the cells were incubated with the Au NPs in the absence of serum, as well as in serum-supplemented media, and NP uptake was quantified as the amount of internalized Au atoms via ICP-MS; *cf.* Figure 3. In agreement with previous studies, NPs were incorporated by cells to a higher extent in the case of serum absence than in serum-supplemented media [23]. As demonstrated in Figure 3, NPs stabilized with collagen were taken up by both cell lines more efficiently than those stabilized with PMA. In serum-free medium, the maximal uptake levels of collagen-coated Au NPs by HeLa and A549 cells measured as the number of incorporated Au atoms amounted to 90,000 and 78,000 ppb, respectively. By contrast, maximal uptake levels of PMA-coated Au NPs by HeLa cells and by A549 cells were 1400 ppb and 850 ppb, respectively. In serum-containing medium, the maximum levels of incorporated Au were 2500 and 4000 ppb for HeLa and A549 cells, respectively. The maximal uptake levels of PMA-coated Au NPs by both HeLa and A549 cells were 400 ppb; *cf.* Figure 3. Note that cells were incubated with the same concentration of Au NPs. As the collagen-coated Au NPs have a larger inorganic core diameter d_c_ than the PMS-coated NPs, cells were exposed to more Au atoms in the case of collagen-coated Au NPs. One Au NP with a core diameter *d*_c_ comprises *N*_Au_ = (4π/3)·(*d*_c_/2)^3^·ρ_Au_/M_Au_ Au atoms, using the density of bulk gold ρ_Au_ =19.32 g/cm^−3^ and the molar mass of one Au atom *M*_Au_ = 196.96 g/mol [27]. Thus, one collagen-coated Au NP in our study represents *N*_Au_(collagen NP)/*N*_Au_(PMA NP) = (*d*_c_(collagen)/*d*_c_(PMA NP))^3^ = (7.4 nm/4.4 nm)^3^ ≈ 4.75-times more Au atoms than one PMA-coated NP. Thus, if cells are incubated with the same number of NPs added, in the case of collagen-coated NPs, cells are incubated with a 4.75-times higher amount of elemental Au than upon incubation with PMA-coated NPs. Even considering this fact, that at equal incubation concentrations of collagen- and PMA-coated Au NPs, cells were incubated with a 4.75-times higher concentration of elemental Au in the case of collagen-coated NPs, the difference in internalized elemental Au is much larger in the case of collagen-coated NPs. Furthermore, in terms of the number of NPs, the collagen-coated NPs were internalized to a much higher extent.

In the case of serum-free incubation conditions, it might be argued that the increase in NP uptake of the collagen- *versus* the PMA-coated NPs was caused by agglomeration of the collagen-coated Au-NPs, causing them to sediment on top of the cells and, thus, to be incorporated faster. However, increased uptake was also observed under serum-containing conditions, in which the collagen-coated Au NPs did not agglomerate to an appreciable extent. Thus, it appears that collagen *versus* PMA surface coating may have a direct effect on the extent to which NPs are taken up, apart from the effect of agglomeration.

### 3.3. Cell Viability upon Incubation with Au NPs

**Figure 4:**
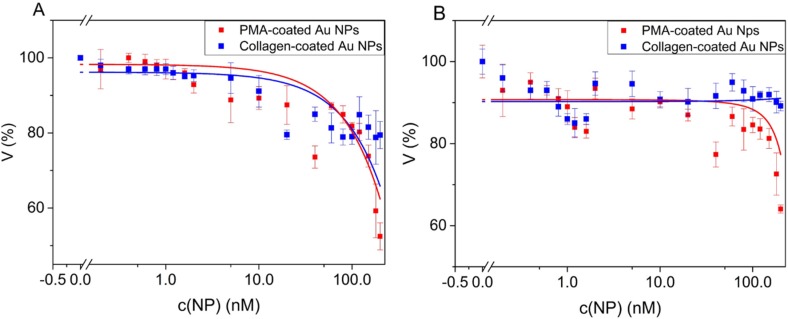
Viability V of HeLa and A549 cells upon incubation with Au NPs. Au NPs were incubated with HeLa (**A**) and A549 (**B**) cells for 4 h in medium with serum. Untreated cells were used as a control (100%). Cell viability was assayed 24 h after incubation with an MTT assay. NP concentrations *c*(NP) between 0.2 nM and 200 nM were tested. The lines between the individual points are merely intended as a guide to the reader; they do not correspond to a mathematical fit.

The potential of Au NPs towards future medical applications can be properly evaluated only if possible toxic effects on cells are taken into consideration. Therefore, we assessed the impact of collagen- and PMA-coated Au NPs on the viability of A549 and HeLa cells. The NPs were incubated with the cells in the presence of serum. The cells were exposed to a range of Au NP concentrations between 0.2 nM and 200 nM. As shown in Figure 4, the collagen-coated Au NPs induced a lower toxic effect than PMA-coated Au NPs, in both cell lines tested. As expected, the most toxic effects were observed at the highest concentration tested (200 nM). Under those conditions, cell viability was reduced to around 80% for collagen-coated Au NPs and around 60% for PMA-coated Au NPs. The higher toxicity of PMA-coated Au NPs could be related to the presence of residual TOAB on the NPs surface [27]. The low toxicity of collagen-coated Au NPs is not surprising, since collagen is a protein that forms an integral part of biological systems. Similar results were found when testing the cytotoxicity of other protein-coated Au NPs, ranging from peptides [28], amino acids [5] to proteins, such as albumin [29,30]. For the direct comparison between collagen- and PMA-coated Au NPs, we applied only short exposure times (four hours). Thus, the reduction in cell viability occurs only at very high NP concentrations. Data from previous studies show that upon prolonged exposure times, the toxic effects of PMA-coated Au NPs (and similar NPs) occur already at lower NP concentrations [15,23,31].

## 4. Conclusions

In this work, we characterized spherical collagen-coated Au NPs with a mean core diameter of 7 nm. These NPs were obtained by following a straightforward one-step synthesis method. Collagen acted as a reducing as well as a stabilizing agent. Internalization and toxicity of collagen-coated Au NPs were tested in A549 and HeLa cells. In contrast to common synthesis protocols, in which Au NPs are first prepared with different, often synthetic, ligands, and then protein coating is achieved by ligand exchange and/or adsorption, here, the Au NPs are directly grown with a native collagen shell. This prevents the presence of any residual synthetic ligand on their surface. The results obtained for the collagen-coated NPs were compared to the data for Au NPs in the same size range, but coated with a synthetic polymer (PMA). NPs stabilized with collagen were taken up by both cell lines more efficiently than those stabilized with PMA, both in the presence and absence of serum. Concentration-dependent toxicity studies further revealed that at short exposure times, collagen-coated Au NPs were less toxic to the cells than PMA-coated Au NPs. Importantly, the collagen-coated NPs, which are internalized to a high degree, exhibit lower toxicity. We speculate that the lack of any synthetic ligands on the surface of collagen-coated NP may be beneficial, whereas in the case of PMA-coated Au NPs, part of the toxicity may originate from residual TOAB fragments due to incomplete ligand exchange. It is also important to note that the coating of the Au NP surface will not remain stable after the NPs have been internalized by cells. This has been demonstrated for both particle types. For Au NPs coated with proteins, it has been shown that a fraction of the proteins may be enzymatically degraded or exchanged with other proteins present in different endosomal compartments [32]. Furthermore, in the case of PMA-coated Au NPs, part of the coating may be replaced *in vitro* as *in vivo* [17]. Still, the results presented here support the notion that the material employed for the original coating of Au NPs plays a role in their biocompatibility and the pattern of internalization. In this context, naturally-occurring macromolecules, such as collagen, may represent a group of interesting stabilizing agents to be used for future biomedical applications.

## Supplementary Materials

Supplementary materials can be accessed at: http://www.mdpi.com/2079-4991/5/3/1418/s1.
