# Supplementary material for: Comparison of the in Vitro Uptake and Toxicity of Collagen- and Synthetic Polymer-Coated Gold Nanoparticles
Source: Nanomaterials (Basel). 2015 Aug 27;5(3):1418–30. doi: 10.3390/nano5031418 (PMC5304636; doi:10.3390/nano5031418)
Supplement: Supplementary file 1 [file nanomaterials-05-01418-s001.pdf]

Supplementary Information

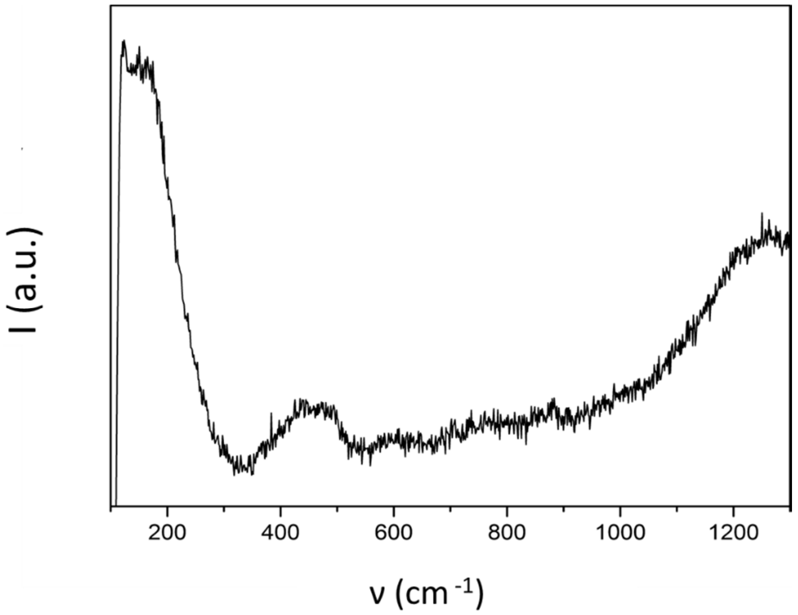

Figure S1. Raman spectrum of collagen-coated Au NPs in which the Raman intensity I is plotted *versus* the wavenumber ν. The SERS spectra were recorded using an Advantage 200A Raman spectrometer (DeltaNu, Laramie, WY, USA). The HeNe laser emitted at 632.8 nm with a power of 3 mW. The spectral resolution of the recorded spectra was ~8 cm^{-1}. The spectrum is the result of the average of 10 accumulations with a 4-s integration time. No indication of Raman bands arising from the presence of collagen at the surface of Au NPs can be seen.

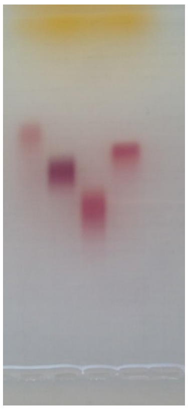

Figure S2. Image under visible light illumination of a 2% agarose gel on which Au NPs have been run with gel electrophoresis according to previously published protocols [1]. The band on the top is due to the Orange-G-containing loading buffer. The bands of the Au NPs can be seen by their red color due to the surface plasmon resonance absorption. The NPs ran from the well seen at the bottom of the image towards the plus pole, which was located on the side of the gel, which is shown at the top of the image. The lanes correspond from left to right to 5-nm core Au NPs with phosphine coating, PMA-coated Au NPs, collagen-coated Au NPs and 10-nm core Au NPs with phosphine coating [1].

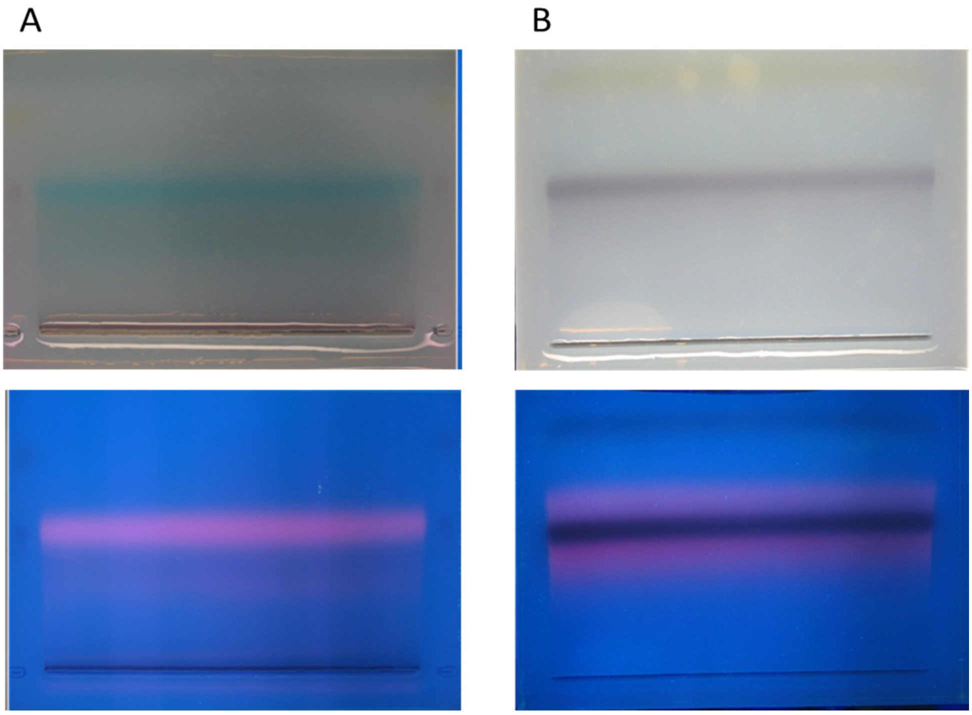

Figure S3. Photographs of 2% agarose gels in which Dy647-labelled Au NPs had been run for 1 h at 100 V and 200 mA according to previously published protocols [1]. The NPs had been loaded in the wells visible at the bottom of the images, and the plus pole towards which the negatively-charged NPs migrated was located on the side of the gels, which is shown at the top of the images. Images under illumination with visible light (top row) and UV light (bottom row) are shown. The band containing the Au NPs was cut out of the gel, and the NPs were extracted. (**A**) Collagen-coated Au NPs. (**B**) PMA-coated NPs.

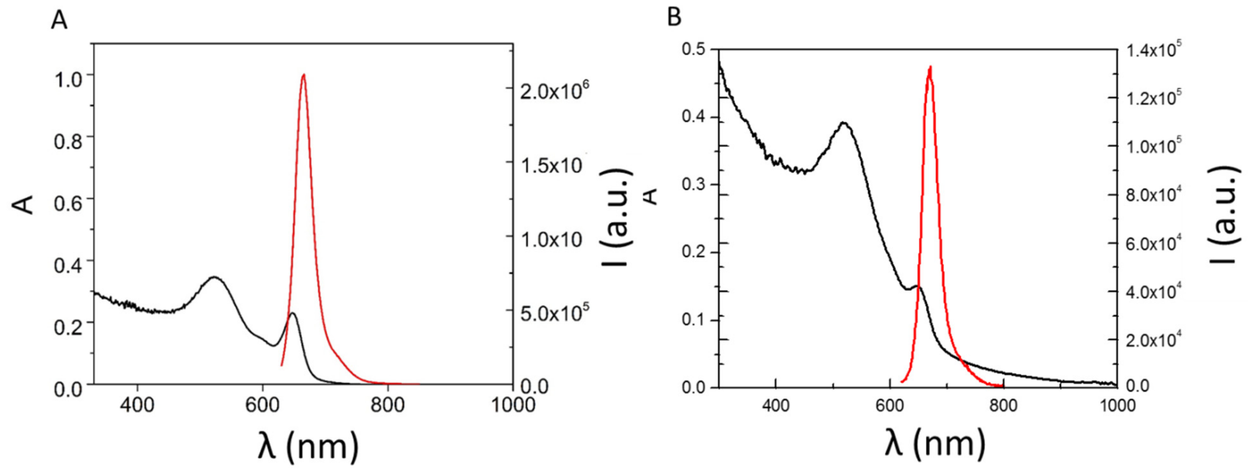

Figure S4. UV-VIS absorption spectra *A*(λ) (in black) and fluorescence emission spectra *I*(λ) (in red) of Dy647-labelled Au NPs as recorded in water. (**A**) Collagen-coated Au NPs. (**B**) PMA-coated Au NPs.

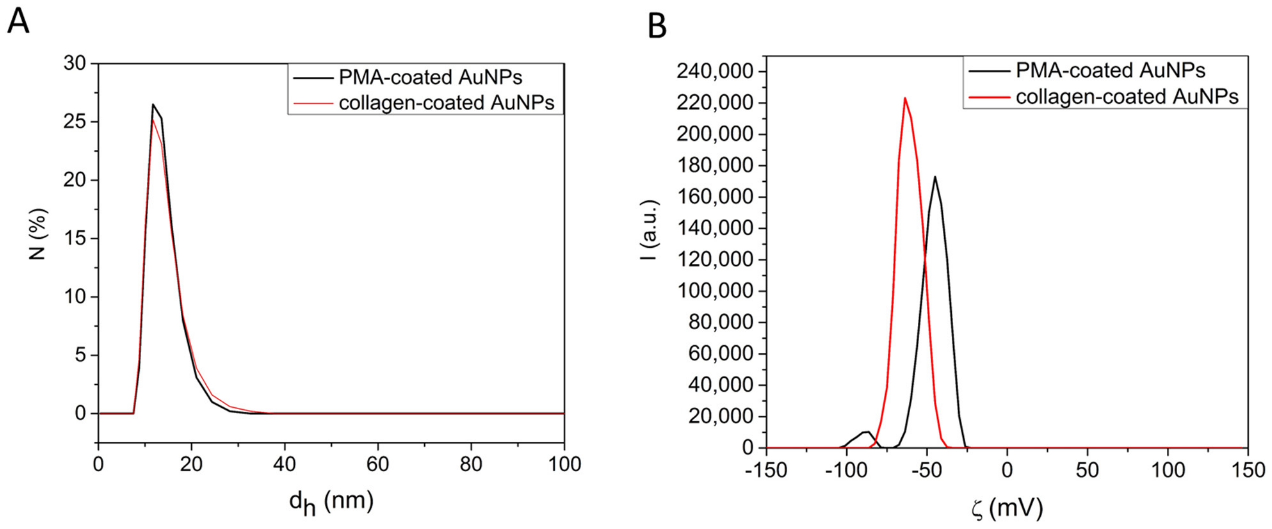

Figure S5. Colloidal properties of collagen- and PMA-coated Au NPs as determined in water. (**A**) Number distribution $N(d_h)$ of the hydrodynamic diameter d_h. The mean hydrodynamic diameters of the collagen- and PMA-coated NPs were determined to be $d_h = 13.5 \pm 0.5$ nm and $d_h = 11.7$ nm, respectively. (**B**) Distribution $I(\zeta)$ of the ζ-potential of collagen- and PMA-coated Au NPs. The mean ζ-potential was determined to be $\zeta = -58.4 \pm 2.5$ mV and $\zeta = -38$ mV for collagen- and PMA-coated Au NPs, respectively.

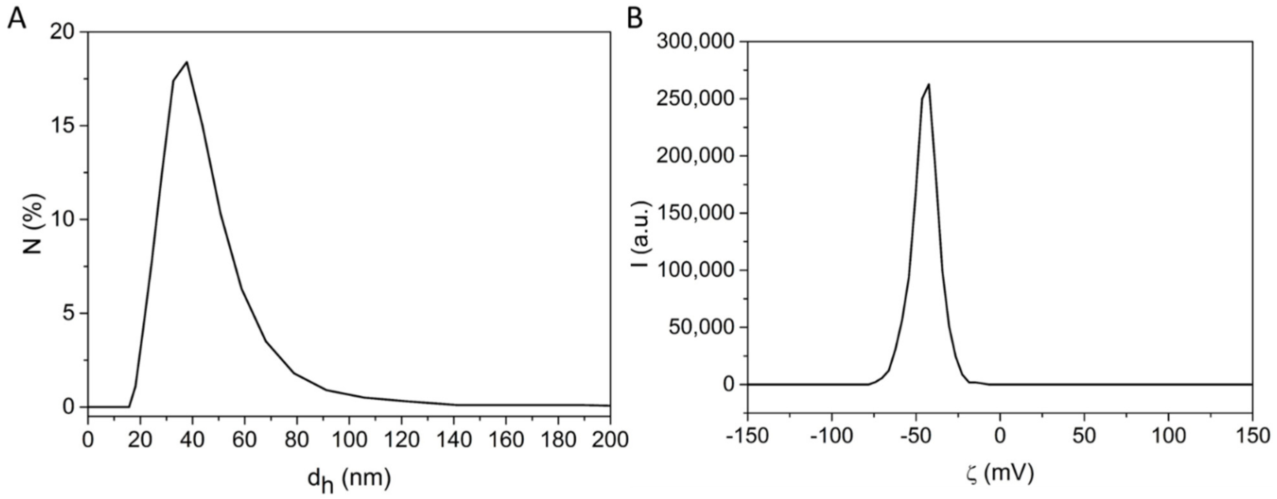

Figure S6. Colloidal properties of Dy647-labelled collagen-coated Au NPs as determined in water. (**A**) Number distribution $N(d_h)$ of the hydrodynamic diameter d_h. The mean hydrodynamic diameters of the collagen-coated NPs were determined to be $d_h = 37.0 \pm 0.5$ nm. (**B**) Distribution $I(\zeta)$ of the ζ-potential of collagen-coated Au NPs. The mean ζ-potential was determined to be $\zeta = -42.4 \pm 3.6$ mV for collagen-coated Au NPs.

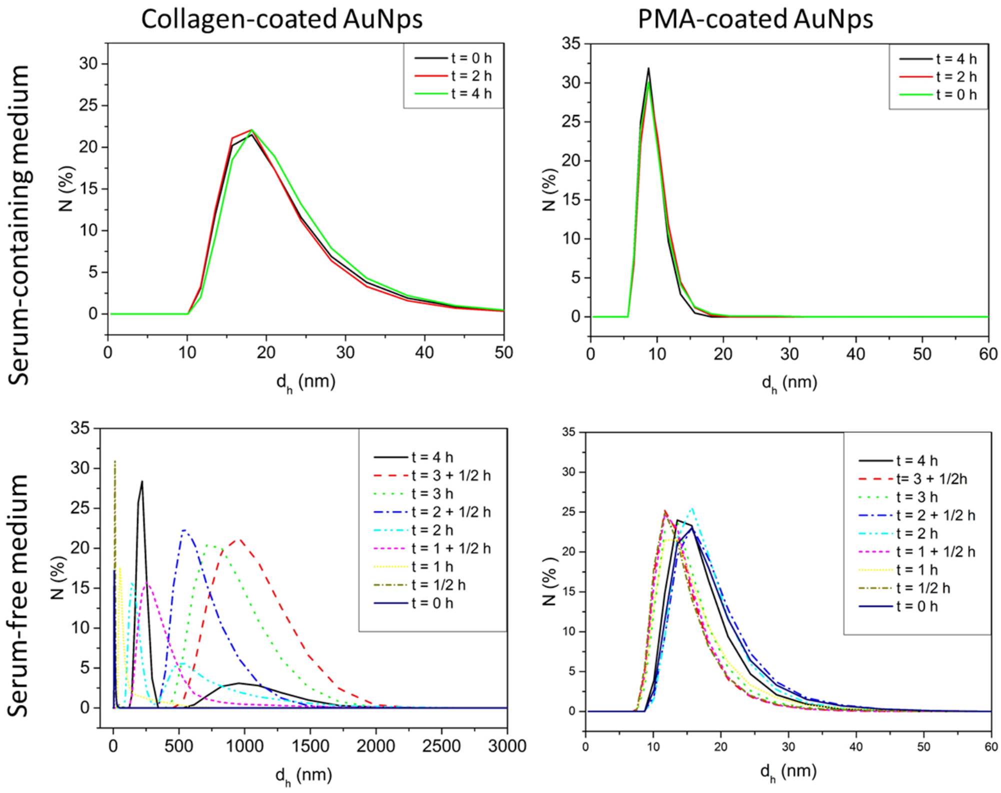

Figure S7. Au NPs (c(NP) ≈ 100 nM) were incubated in serum-free and serum-containing DMEM medium and the number distribution $N(d_h)$ of the hydrodynamic diameter d_h was measured with DLS at different incubation times *t*. (**A**) Collagen-coated Au NPs. (**B**) PMA-coated Au NPs.

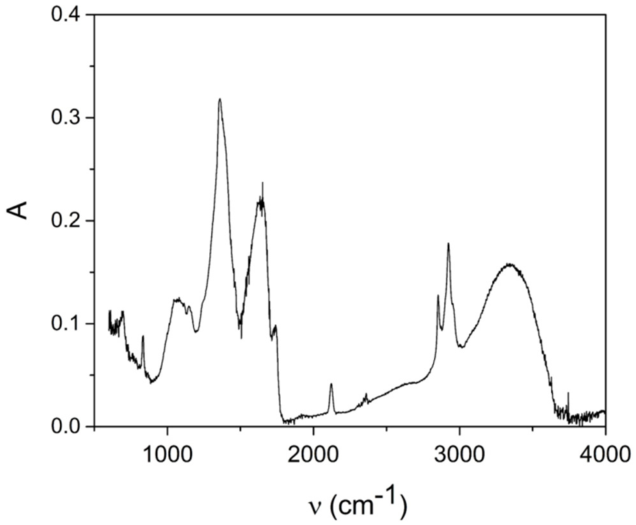

Figure S8. FTIR spectrum of collagen-coated Au NPs (Jasco FTIR Spectrometer). The molecular fingerprint of collagen can be recognized in the spectrum. The presence of ν(C–H) bands at 2920 and 2850 cm^{-1}, ν(C=O) vibrations at 1740 cm^{-1}, the presence of an amide I band specific for proteins, as a result of ν(C=O) vibrations, C–C–N molecular bond deformations, out-of-phase ν(C–N) and in-plane δ(N–H) vibrations and the amide III band, corresponding to ν(C–N) and δ(N–H) vibrations, are well represented, leading to the conclusion that collagen is adsorbed at the surface of Au NPs [2,3].

References

1. Pellegrino, T.; Sperling, R.A.; Alivisatos, A.P.; Parak, W.J. Gel electrophoresis of gold-DNA nanoconjugates. *J. Biomed. Biotechnol.* **2007**, *2007*, 26796.
2. Kumari, K.; Singh, P.; Mehrotra, G.K. A facile one pot synthesis of collagen protected gold nanoparticles using Na–malanodialdehyde. *Mater. Lett.* **2012**, *79*, 199–201.
3. Sun, Y.; Sun, L.; Zhang, B.; Xu, F.; Liu, Z.; Guo, C.; Zhang, Y.; Li, Z. Type I collagen-mediated synthesis of noble metallic nanoparticles networks and the applications in Surface-Enhanced Raman Scattering and electrochemistry. *Talanta* **2009**, *79*, 562–569.

© 2015 by the authors; licensee MDPI, Basel, Switzerland. This article is an open access article distributed under the terms and conditions of the Creative Commons Attribution license (http://creativecommons.org/licenses/by/4.0/).
